# Locoregional Control and Survival in Children, Adolescents, and Young Adults With Localized Head and Neck Alveolar Rhabdomyosarcoma—The French Experience

**DOI:** 10.3389/fped.2021.783754

**Published:** 2022-02-04

**Authors:** Roxane Machavoine, Sylvie Helfre, Valérie Bernier, Stéphanie Bolle, Julie Leseur, Nadège Corradini, Angélique Rome, Anne-Sophie Defachelles, Sophie Deneuve, Sophie Bernard, Pierre Fayoux, Richard Nicollas, Michel Mondain, Romain Luscan, Françoise Denoyelle, François Simon, Natacha Kadlub, Fréderic Kolb, Jean-François Honart, Daniel Orbach, Véronique Minard-Colin, Antoine Moya-Plana, Vincent Couloigner

**Affiliations:** ^1^Department of Pediatric Oto-Rhino-Laryngology, Hôpital Necker-Enfants Malades, APHP, Paris, France; ^2^Department of Radiation Oncology, Institut Curie, Paris, France; ^3^Département Universitaire de Radiothérapie-Curiethérapie, Institut de Cancérologie de Lorraine, Vandœuvre-lès-Nancy, France; ^4^Department of Radiation Oncology, Gustave Roussy, Université Paris-Saclay, Villejuif, France; ^5^Service de Radiothérapie, Centre Eugène Marquis, Rennes, France; ^6^Service d'Oncologie Pédiatrique, Institut d'Hématologie et d'Oncologie Pédiatrique, Hospices Civils de Lyon, Lyon, France; ^7^Pediatric Oncology Department, Hôpitaux Universitaires de Marseille Timone, AP-HM, Marseille, France; ^8^Department of Pediatric Oncology, Centre Oscar Lambret, Lille, France; ^9^Service d'Oto-Rhino-Laryngologie, Centre Léon Bérard, Lyon, France; ^10^Department of Pediatric Oto-Rhino-Laryngology, Hôpital Robert Debré, APHP, Paris, France; ^11^Department of Pediatric Oto-Rhino-Laryngology, Hôpital Jeanne de Flandre, CHRU Lille, Lille, France; ^12^Department of Pediatric Oto-Rhino-Laryngology, Hôpitaux Universitaires de Marseille Timone, AP-HM, Marseille, France; ^13^Service d'Oto-Rhino-Laryngologie, CHU Montpellier, Montpellier, France; ^14^Department of Pediatric Maxillo-Facial and Plastic Surgery, Hôpital Necker-Enfants Malades, APHP, Paris, France; ^15^Plastic Surgery Department, Gustave Roussy, Université Paris-Saclay, Villejuif, France; ^16^SIREDO Oncology Center (Care, Innovation and Research for Children and AYA with Cancer), PSL Research University, Institut Curie, Paris, France; ^17^Department of Pediatric and Adolescent Oncology, INSERM 1015, Gustave Roussy, Université Paris-Saclay, Villejuif, France; ^18^Department of Oto-Rhino-Laryngology, Gustave Roussy, Université Paris-Saclay, Villejuif, France

**Keywords:** alveolar rhabdomyosarcoma (ARMS), head and neck neoplasm, children, neck dissection, survival

## Abstract

**Introduction:**

The head and neck (HN) are the most frequent sites of pediatric rhabdomyosarcoma (RMS). Alveolar RMS (ARMS) represents ~20% of all RMS cases and frequently spread to lymph nodes (LNs). The aim was to report locoregional control, event-free survival (EFS), and overall survival (OS), according to clinical and pathological features, LN staging, and treatment modalities.

**Methods:**

The study included all patients prospectively enrolled in EpSSG RMS 2005 study under 21 years of age with localized HN ARMS and diagnosed between 2005 and 2016 in France. Medical data including imaging, surgical report, and radiation therapy planes were analyzed.

**Results:**

Forty-eight patients (median age 6 years; range 4 months−21 years), corresponding to 30 parameningeal and 18 non-parameningeal ARMS, were included. There were 33 boys (69%). Tumor locations included the following: orbit (*n* = 7) among which four cases had bone erosion, paranasal sinuses and nasal cavity (*n* = 16), deep facial spaces (*n* = 10), nasolabial fold (*n* = 8), and other non-parameningeal HN sites (*n* = 7). A fusion transcript of PAX3-FOXO1 or PAX7-FOXO1 was expressed in 33 of the 45 cases (73%) with molecular analysis. At diagnosis, 10 patients had primary resection of the primary tumor (PRPT) (none with microscopic complete resection) and 9 had LN staging. After induction chemotherapy, 26 patients (54%) had secondary resection of the primary tumor (SRPT) and 13 patients (27%) had cervical LN dissection. A total of 43 patients (90%) were treated with radiation therapy.

With a median follow-up of 7 years (range 2–13 years), 5-year OS and EFS were 78% (95% CI, 63–88%) and 66% (95% CI, 51–78%), respectively. We observed 16 events (10 deaths): 4 local, 4 regional, 1 local and regional, and 7 metastatic. In univariate analysis, OS was only superior for patients under 10 years of age (*p* = 0.002), while *FOXO1*-negative ARMS, SRPT for parameningeal ARMS, and LN surgery were associated with significantly better EFS.

**Conclusion:**

Our study confirms a better outcome for fusion-negative ARMS and ARMS in children under 10 years. Moreover, LN surgery and SRPT of parameningeal tumor may improve EFS of ARMS. Larger studies are needed to confirm our findings.

## Introduction

Rhabdomyosarcoma (RMS) represents about 2–5% of childhood and adolescent cancers (1, 2), with ~40% arising in the head and the neck (3–5). Several RMS histologic subtypes can be distinguished. The two most prevalent ones are embryonal rhabdomyosarcoma (ERMS) that has an intermediate prognosis and alveolar rhabdomyosarcoma (ARMS) that represents about 20–25% of RMS and has a poorer prognosis (6–8). ARMS is proportionately more common than ERMS in children over 10 years old (9). More than 80% of ARMS express fusion transcripts (FTs) between the *FOXO1* and *PAX3* or *PAX7* genes (10, 11). These genetic *FOXO1* anomalies are associated with a poorer prognosis (12). The treatment of these high-risk tumors includes systemic chemotherapy associated with local treatment that may rely on surgery, radiotherapy, or a combination of both. ARMS spreads rapidly locally but also by lymphatic and hematogenous routes. The most frequent extension sites are lymph nodes (LNs), lungs, and bone marrow (13). About half of the patients treated for localized ARMS undergo a relapse (14). The 5-year overall survival (OS) of patients with head and neck ARMS (HN-ARMS) ranges from 35% (15) to 80% (16). To evaluate the prognosis value of clinical and pathological features and the impact on outcome of LN staging and locoregional therapies, we reviewed all patients <21 years with localized ARMS treated in France in the prospective EpSSG RMS 2005 study.

## Patients and Methods

### Population

This multicenter study included all French patients prospectively enrolled in the EpSSG RMS 2005 protocol under 21 years of age with localized HN-ARMS diagnosed between 2005 and 2016 (17). Patient's consent or his/her legal representative's was collected. Analyses were performed on the data derived from EpSSG RMS 2005 study. Additional data, particularly those concerning modalities of LN staging, surgery, and radiotherapy, were retrieved from medical center files. Based on initial RMS2005 criteria, pathologists should consider ARMS diagnosis when tumor showed any focal alveolar pattern histology. However, since RMS2005 trial ran from 2005 to 2016, it was next recommended in the pathologist community to consider ARMS if alveolar pattern was predominant. The location of the primary tumor was determined by imaging at the time of diagnosis and classified into three sites: orbit, non-parameningeal (non-PM), and PM sites. For PM tumors, a cranial nerve palsy, a skull base erosion, an intracranial extension, and the presence of tumor cells in the cerebrospinal fluid (CSF) were systematically looked for at diagnosis.

### Staging

Initial staging was established according to the TNM (18) and Intergroup Rhabdomyosarcoma Study Group (IRSG) (both surgical-pathologic grouping and staging systems) (19) classifications. LN involvement was assessed by initial computed tomography (CT), magnetic resonance imaging (MRI), or positron emission tomography–computed tomography (PET-CT). For ARMS, systematic LN evaluation was further recommended by cytological or pathological analysis of nodal samples. Any distant metastasis at the time of diagnosis was researched by technetium bone scan or PET CT, bone marrow biopsy, and aspiration.

### Treatment

Treatment protocol EpSSG RMS 2005 has been previously reported (20). All localized ARMS were considered as high-risk RMS. For N0 ARMS, patients were randomized to receive either standard IVA (ifosfamide, vincristine, dactinomycin) or IVADo (ifosfamide, vincristine, dactinomycin, and doxorubicin)/IVA for a total of 9 courses. Patients with tumor in remission after 9 courses, surgery, and/or radiation therapy (RT), were randomly assigned to stop treatment or receive maintenance chemotherapy [six 28-day cycles of intravenous (i.v.) vinorelbine and oral cyclophosphamide]. For N1 ARMS, patients received intensified induction chemotherapy (IVADo/IVA) and additional maintenance chemotherapy with systematic local treatment to primary and nodal sites. The total duration of chemotherapy was 50 weeks.

### Surgical Strategy

In case of primary resection of the primary tumor (PRPT) (resection of the primary site of the tumor prior to any other treatment), the status of surgical margins was categorized from R0 to R2 (R0: macroscopically and microscopically complete resection; R1: microscopically incomplete resection; R2: macroscopically incomplete resection), and the quality of tumor resection was defined using the IRSG surgical-pathologic grouping system (20). When secondary surgery was performed after induction chemotherapy, 3 types of procedure were distinguished: extensive resection of the initial tumor extensions (“ghost surgery”), resection of the residual [both considered as secondary resection of the primary tumor (SRPM)], or exploration. As in the case of PRPT, the status of surgical margins was ranked from R0 to R2. The necessity of a reconstruction and its type (pedicled flap, free flap) and the transient or definitive need for a tracheostomy or gastrostomy were noted. Mutilating surgery was defined by the presence of a permanent postoperative cranial nerve paralysis and by the necessity of infratemporal fossa, maxillary or mandibular resection. Concerning LN surgery, 4 strategies were distinguished: sentinel LN biopsy (SLNB), suspicious node excision, LN sampling, and LN dissection. After pathological analysis, resected LNs were divided into healthy ones (pN0) and pathological ones (pN1).

### Radiation Therapy

Different types of RT, brachytherapy or external radiation therapy [proton beam therapy (PBT) or intensity-modulated radiation therapy (IMRT)], were performed. RT target could concern the initial or residual tumor volume, LN chains, and the transit pathway from the tumor primary site to the nearest LN chain. In the RMS 2005 protocol, reduction of radiation dose for patients who underwent secondary surgery was not planned.

### Statistics

Follow-up was defined as the time between diagnosis and the patient's last visit or death date. Relapse was defined by cancer recurrence after a period of complete remission: it could be local, regional (nodal), or metastatic, regardless of the initial T and N status. Progression was defined by tumor volume increase or the occurrence of new lesions during treatment. OS was defined as the time between diagnosis and date of last visit or death. Event-free survival (EFS) was defined as the time between diagnosis and occurrence of an event such as relapse, progression, or death from any cause. In case of RT, local or nodal relapses were defined as in-field, marginal, or out-of-field. Univariate analysis and correlation between two qualitative variables were estimated with chi-square test and Fisher's exact test. Survival curves were calculated by the Kaplan–Meier method. The 5-year OS and the 5-year event-free percent survival were expressed with their confidence interval. The log-rank test was used for univariate analysis of survival data. For all statistical tests used, results were considered significant for a *p* ≤ 0.05. Data were analyzed using GraphPad Prism software (version 8.00 GraphPad Software, La Jolla, CA, USA; www.graphpad.com).

## Results

### Patient Characteristics

**Figure 1:**
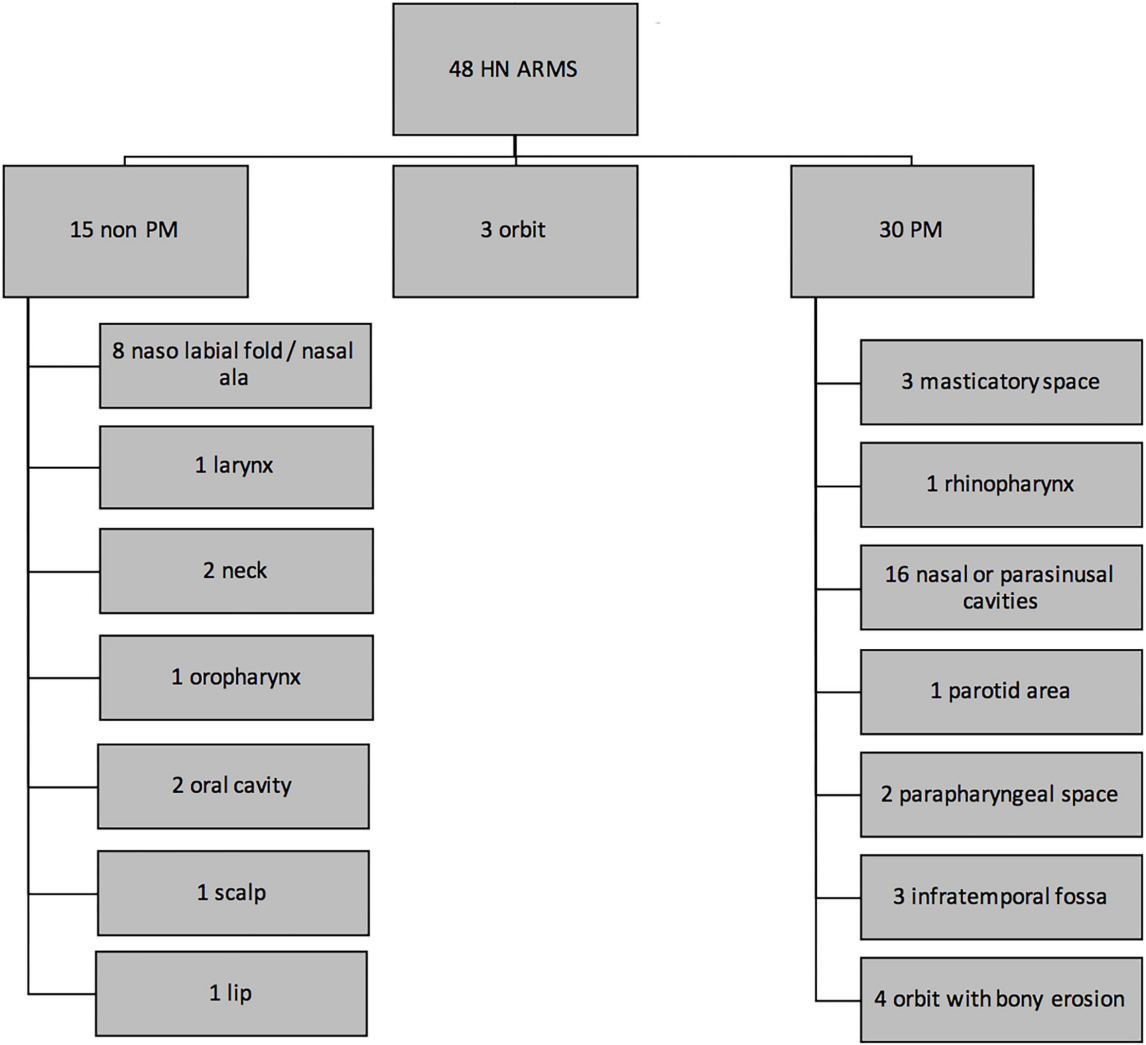
Locations of the 48 head and neck alveolar rhabdomyosarcomas (HN-ARMSs).

Forty-eight patients diagnosed between 2005 and 2016 in France were included in the analysis. Median age was 6.2 years (range 4 months−20.3 years) (Table 1). Fifteen patients had a non-PM and 30 a PM HN-ARMS of which locations are detailed in Figure 1. Three patients had an orbital tumor without bone erosion. Of the 30 patients with PM tumor, 21 (70%) had a skull base erosion, 6 (20%) had an intracranial tumor extension, and 10 (33.3%) had a cranial nerve palsy at the time of diagnosis. The tumor expressed a *PAX3/PAX7-FOXO1* FT in 33 patients over 45 tested (73%). Thirty-one patients were N0 (65%) and 17 N1 ARMS. There was no significant difference in LN status (N0 or N1) at diagnosis depending on initial tumor extension (T status) (*p* = 0.544).

**Table 1:** Patient characteristics.

		***n***	**%**
**Age**			
	0–12 months	3	6.25%
	13 months−10 years	30	62.5%
	>10 years	15	31.25%
**Sex**			
	Female	15	31.25%
	Male	33	68.75%
**Histology**			
	ARMS	46	95.8%
	Solid ARMS	2	4.2%
**PAX3/PAX7-FOXO1 FT expression**			
	Yes	33	68.75%
	No	12	25%
	Investigation not done	3	6.25%
**Tumor stage at diagnosis**			
	T1	21	43.75%
	T2	27	56.25%
**Tumor size at diagnosis**			
	a <5 cm	25	52.08%
	b >5 cm	21	43.75%
	x: unavailable	2	4.17%
**Nodal stage at diagnosis**			
	N0	31	64.6%
	N1	17	35.4%
**IRS group**			
	I	0	0%
	IIa	3	6.2%
	IIb	0	0%
	IIc	0	0%
	IIIa	38	79.2%
	IIIb	7	14.6%
	IV	0	0%
**Tumor location**			
	Orbit	3	6.25%
	Non-parameningeal	15	31.25%
	Parameningeal	30	62.5%

*ARMS, alveolar rhabdomyosarcoma*.

### Lymph Node Staging

PET-CT was performed for 34/48 patients (71%). Twenty-eight patients had negative LN on PET-CT. Among them, three had abnormal LN on CT and/or MRI. Among the 6 patients with abnormal LN fixation on PET-CT, 2 patients were considered N0 (1 pN0 after biopsy and 1 for whom LNs were not visualized on MRI and were not considered for LN staging). None of these 2 patients had LN relapse. The 4 other patients received a nodal treatment (radiotherapy only *n* = 2 or combination of LN dissection and RT *n* = 2). Among these last 4 patients, 3 were alive on first complete remission and 1 deceased after displaying a nodal relapse. Nine patients underwent initial cytological or pathological assessment of LN areas (3 because of abnormal LN on conventional imaging, 2 because of abnormal LN fixation on PET-CT, 1 because of anomalies on both conventional imaging and PET-CT; in the last 3 cases, LN cytological or pathological assessment was performed despite normal CT/MRI/PET-CT results): 4 underwent LN fine-needle aspiration (of which 3 were pN1), 3 had LN biopsy (1 pN1), 1 had sentinel node biopsy (pN0), and 1 had LN dissection (pN1). Among the 17 patients classified as N1: 10 were on clinical evaluation and conventional imaging (CT and/or MRI), 2 were on PET/CT only, and 5 were confirmed by pathological examination of LN. There was no correlation between the initial N status and the performance of PET-CT at diagnosis (*p* = 0.201). The PET-CT positive predictive and negative predictive values for proven pathological nodal disease (pN1) or nodal relapse were 75 and 50%, respectively.

### Response to Chemotherapy

After induction chemotherapy, out of the 42 patients with evaluable disease, 6 (14.3%) had a complete response, 10 (23.8%) had a very good partial response, 16 (38.1%) had a partial response, 6 (14.3%) had a minor partial response, 3 (7.1%) had tumor stability, and 1 (2.4%) had tumor progression.

### Surgical Strategy

For 32 patients (67%), diagnosis of ARMS was made by surgical biopsy. For 4 patients, the diagnosis was made by ultrasound (US)-guided “tru-cut®” biopsy. PRPT was performed in 10 patients: 7 resections were macroscopically incomplete (R2) (70%) and 3 were microscopically incomplete (R1) (30%). For 1 patient, diagnosis was made on pathological analysis of an LN dissection. One diagnosis of ARMS was made on fine-needle aspiration.

**Figure 2:**
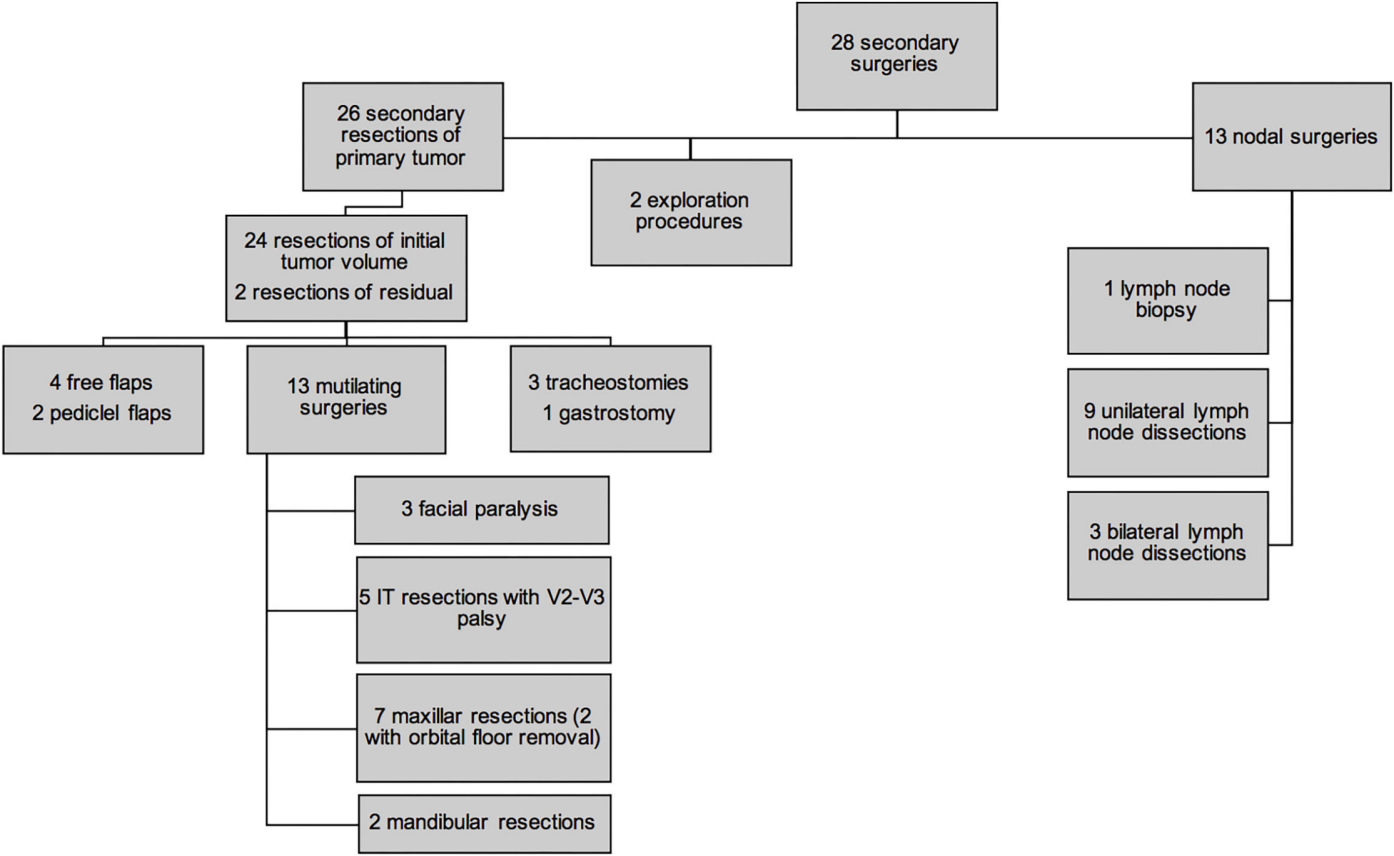
Local and nodal strategy for secondary surgery. IT, infratemporal; V2–V3: maxillary (V2) and mandibular (V3) nerves.

**Figure 3:**
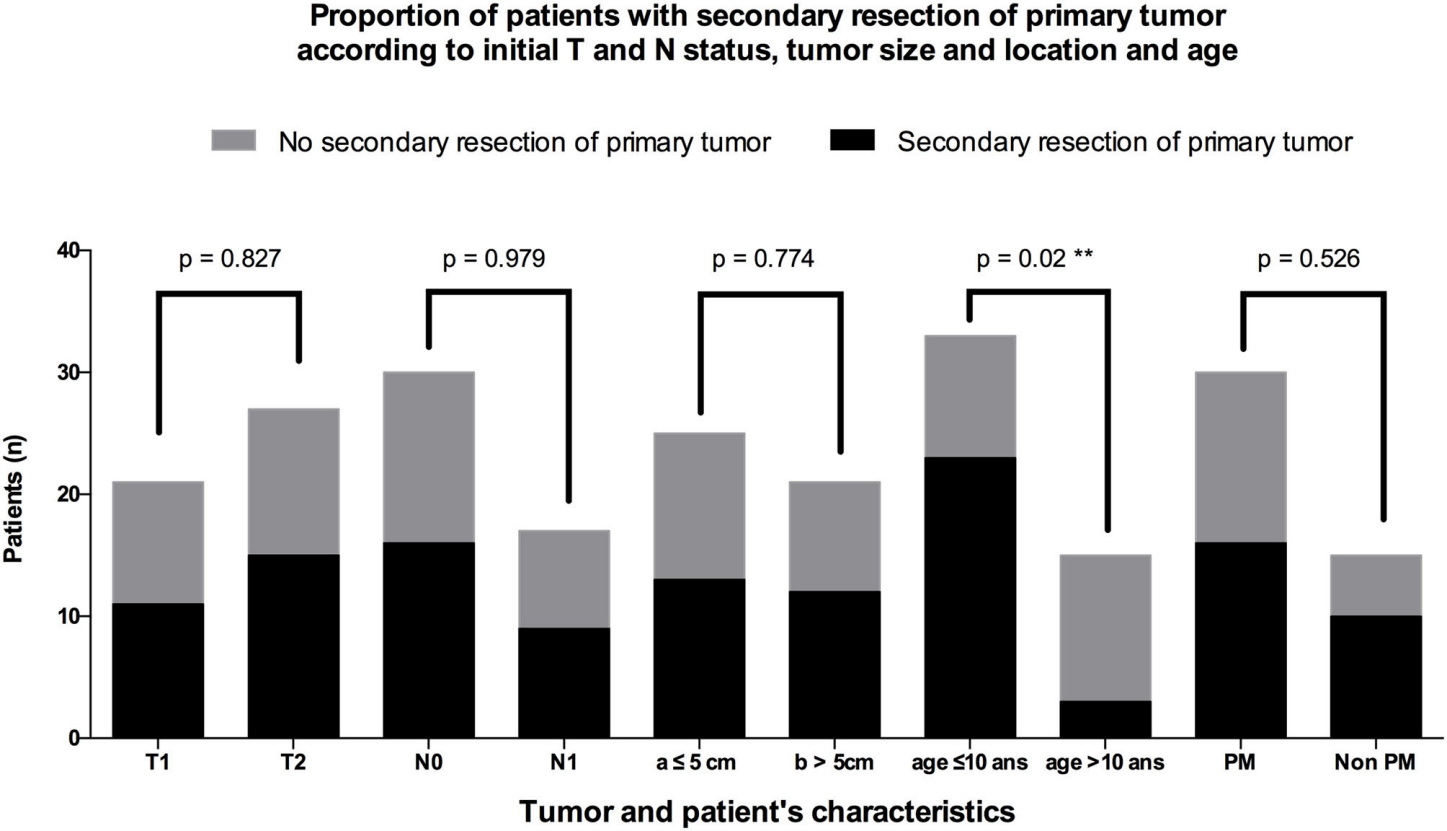
Repartition of operated and non-operated patients according to the initial T and N status, tumor size and location, and patient age.

Twenty-eight (58.3%) patients underwent secondary surgery after chemotherapy (Figure 2). Twenty-six were SRPT (10 non-PM, 16 PM): 24 had extensive surgery with the aim of removing initial tumor volume (“ghost surgery”), and 2 were limited to the residual mass. In 1 case of nasal sinus tumor, secondary surgery only consisted of surgical exploration with tumor mapping. In case of nasal ala ARMS, no tumor remnant was identified during surgery: in Figure 2, this case was also categorized as a surgical exploration. None of the 3 patients with localized orbital tumor underwent secondary surgery. The 26 SRPT were 2 total parotidectomies, 1 with sacrifice of the facial nerve and the other extended to the masseter and the zygomatic and malar region; 5 resections of the nasolabial fold extended to the nasal ala, cheek, upper lip, lower turbinate, and nasal bones, and in 4 cases extended to the maxillary bone at the level of the piriform aperture; 1 revision surgery in the anterior neck region with resection of the hyoid bone; 1 partial glossectomy; 1 revision of scalp resection; 1 labiectomy; 5 total maxillectomies; 2 external temporal fossa resections; 2 spheno-ethmoidal surgeries, one of which was limited to the excision of the residual tumor; 5 infratemporal fossa resections, two of which were extended to the parotid with 1 postoperative facial paralysis; 1 arytenoidectomy. Thirteen SRPTs were classified as mutilating according to the criteria as defined in the *Patients and Methods* section: 3 facial paralysis, one after parotidectomy with sacrifice of the facial nerve and two after laterally extended resection of the infratemporal fossa; 5 infratemporal fossa resections and 7 maxillary resections with orbital floor removal in 2 cases. Four resections were reconstructed by free flap (2 *latissimus dorsi* muscle flap, 1 scapular dorsal flap, 1 thoracodorsal artery perforator free flap) and two by a pedicle flap (1 temporalis muscle flap and 1 Abbe flap). Four surgeries required a transient tracheotomy and 1 patient required a transient gastrostomy after arytenoidectomy due to choking. Of the 26 patients who underwent SRPT, 10 patients (38.5%) had microscopically negative margins (R0). For 14 patients (54%), the margins were microscopically positive (R1). In 2 cases (7.7%), the excision was macroscopically incomplete (R2). The residual lesions were located in the cavernous sinus and in the temporomandibular joint next to the resection margins in the other. There were more patients under 10 years of age who underwent an SRPT than patients older than 10 years of age (*p* = 0.002) (Figure 3). There was no difference between patients who underwent SRPT and patients who did not according to initial T and N status, tumor size, and tumor location.

Thirteen cervical LN surgeries were performed (7 N0 patients, 6 N1 patients): 9 unilateral and 3 bilateral LN dissections, and 1 single retropharyngeal LN resection. Among these 13 patients, 8 had a PM and 5 non-PM ARMS. None of the 3 patients with a tumor limited to the orbit had secondary LN surgery. In 4 patients (30.77%), histological analysis revealed LN metastases (3 PM and 1 non-PM ARMS). No N0 patient at the time of diagnosis was pN1. Among the 19 patients with pathological LN assessment (initial staging and/or secondary nodal surgery), 1 patient displayed an LN relapse vs. 4 patients among the 29 patients without LN assessment (*p* = 0.635).

### Radiation Therapy

Forty-three patients (90%) had RT, including 42 external beam RT and 1 iridium-192 brachytherapy. Among the 42 patients treated with external beam RT, 33 received IMRT and 5 PBT. For 4 patients, the external RT technique was not specified. Five patients did not receive RT treatment due to their very young age and/or complete second surgery. Their median age was 14 ± 18 months (minimum 4 months; maximum 48 months). For the 42 patients treated with external RT, 25 irradiation fields targeted only the primary tumor site, whereas 16 fields included the primary tumor site and LN chains. For the patient who displayed a nodal progression after secondary surgery and before the end of chemotherapy, the irradiation field targeted only LN areas. Of the 41 irradiation fields targeting the primary tumor, 32 included the initial tumor volume and 5 concerned the residual tumor volume after chemotherapy and/or surgery. The median radiation dose of external RT was 50.4 Grays ± 4.5 (range 38.6–56) and 44.8 Grays ± 5.2 (range 41.4–54.4) for primary tumor site (data available *n* = 38) and nodes (data available *n* = 15), respectively.

### Events

For alive patients, the median follow-up was 7.4 years (standard deviation 3.2 years; minimum 2.2 years; maximum 13.2 years). An event occurred in 16 patients (33.3%) (Table 2). All were related to disease failure: 3 patients had local relapse, 1 local and LN relapse, 3 LN relapse, 6 metastatic relapse, and 1 local relapse associated with metastases. A local progression was observed in 1 patient and in another one who initially had no adenopathy on imaging (N0), LN metastases appeared during chemotherapy. No in-transit LN metastasis was observed. In total, local, regional LN, and metastatic relapse/progression represented 40, 31, and 44% of events, respectively. The median delay between diagnosis and event was 20 ± 12 months (range 7–45 months). Ten patients (62%) died, and the 6 other ones were disease-free at their most recent visit. Among the 10 patients who displayed a local or nodal event, 5 had a PM ARMS and 5 a non-PM ARMS (Table 3).

**Table 2:** Details of events according to the tumor location.

	**Events**						
	**Local**	**Nodal**	**Local and**	**Metastatic**	**Local and**	Progressions^*^	**All**
	**relapses**	**relapses**	**nodal relapses**	**relapses**	**metastatic relapses**		**(*n* = 16)**
Orbit (*n* = 3)	0/3 (0%)	0/3 (0%)	0/3 (0%)	0/3 (0%)	0/3 (0%)	0/3 (0%)	0/3 (0%)
Non PM (*n* = 15)	0/15 (0%)	2/15 (13%)	1/15 (7%)	2/15 (13%)	1/15 (7%)	1/15 (7%)	7/15 (47%)
PM (*n* = 30)	3/30 (10%)	1/30 (3%)	0/30 (0%)	4/30 (13%)	0/30 (0%)	1/30 (3%)	9/30 (30%)
All (*n* = 48)	3/48 (6%)	3/48 (6%)	1/48 (2%)	6/48 (13%)	1/48 (2%)	2/48 (4%)	16/48 (33%)

* *One local and one nodal progression*.

*PM, parameningeal*.

**Table 3:** Details of local and nodal treatments for the 10 patients who displayed an event.

**Age (years and months)**	**Tumor location**		**TNM**	**Secondary surgery**		**Radiation therapy (RT)**		**Event**	**Relapse location in relationship with RT field**	**Last status**
				**Local (margins)**	**Nodal (pN)**	**Local RT**	**Nodal RT**			
3y 2m	Non-PM	Arytenoid	T2N0	Initial tumor resection (R1)	–	Initial tumor volume	–	Nodal relapse	Out-field	Complete remission
8m	Non-PM	Nasal ala	T1N0	Initial tumor resection (R1)	–	–	–	Local and nodal relapse	–	Complete remission
7y 4m	Non-PM	Nasal ala	T1N1	–	Unilateral lymph node dissection (pN1)	Initial tumor volume	Unilateral cervical	Nodal relapse	Marginal	Dead
13y	PM	Maxillary sinus	T2N0	–	–	Initial tumor volume	–	Local relapse	In-field	Dead
1y 9m	PM	Orbit with bony erosion	T2N0	–	–	Initial tumor volume	–	Local relapse	In-field	Complete remission
19y 7m	PM	Maxillary sinus	T2N0	–	–	Residual tumor volume	–	Local relapse	In-field	Complete remission
4m	Non-PM	Retroauricular scalp	T1N0	Initial tumor resection (R0)	–	–	–	Local and metastatic relapse	–	Dead
3y 10m	Non-PM	Upper lip	T1N0	Initial tumor resection (R1)	–	–	Unilateral cervical (after nodal progression)	Nodal progression (before end of induction chemotherapy)	Nodal progression before RT (no local or nodal relapse after RT)	Complete remission
13y 4m	PM	Ethmoidal sinus	T2N1	–	–	Initial tumor volume	Unilateral cervical and retropharyngeal	Nodal relapse	In-field	Dead
20y 9m	PM	Orbit with bony erosion	T2N0	–	–	Initial tumor volume	–	Local progression	In-field (patient refused to receive the initially planned radiation dose)	Dead

*PM, parameningeal; RT, radiation therapy*.

Among the 5 patients who had a nodal event either a relapse (*n* = 4) or LN progression (*n* = 1), 2 were N1 at diagnosis and 3 were N0; 4 had a PET-CT at diagnosis, one of which showed signs suggestive of LN metastasis. The 2 N0 patients who displayed a nodal relapse had undergone a PET-CT at diagnosis. One patient (7.7%) over the 13 who underwent a nodal secondary surgery presented with a nodal relapse vs. 4 (11.4%) over the 35 patients who did not. No patient who had a secondary nodal surgery presented with metastatic relapse compared to 7 patients out of 35 (20%) without secondary LN surgery. Radiation field targeted the primary tumor in 7 cases, both the primary tumor and LN chains in 2 cases, and only LN chains in 1 case (patient with nodal progression). There were 5 in-field relapses (one of which was probably due to patient's refusal to receive the full initially planned radiation dose), 1 nodal marginal relapse, and 1 nodal out-field relapse. Among the 5 patients who did not receive RT, 1 had a local and nodal relapse and another one died of a local and metastatic relapse.

### Univariate Analysis of Relapses According to Initial Characteristics and Treatment

Of the 16 patients who displayed an event, none had an initial tumor localized to the orbit, 7 had a non-PM tumor, and 9 had a PM tumor (Table 4). The median age of patients with relapse was 10.2 ± 7.3 years (minimum 4 months; maximum 20.8 years). Of these 16 patients, 14 had a tumor expressing an FT, and for the other two, these data were not available. No patient whose tumor did not express FT had relapse or progression. Among the 16 tumors that relapsed, 6 were T1 and 10 were T2 at diagnosis; 11 were N0 and 5 were N1. In univariate analysis, age over 10 years and tumor expression of a PAX3/PAX7-FOXO1 FT were significant risk factors of event (*p* = 0.048 and *p* = 0.004, respectively).

**Table 4:** Univariate analysis of events according to patients and tumor characteristics and treatment for the 48 HN-ARMSs.

		***n***	**%**	**Complete remission without event**	**Event**	***p***
Age						**0.048**
	0–10 years	33	6.25%	25	8	
	>10 years	15	31.25%	7	8	
Sex						1.000
	Female	15	31.25%	10	5	
	Male	33	68.75%	22	11	
Histology						0.546
	ARMS	46	95.83%	30	16	
	Solid ARMS	2	4.17%	2	0	
PAX3/PAX7-FOXO1 FT expression						**0.004**
	Yes	33	68.75%	19	14	
	No	12	25.00%	12	0	
	Investigation not done	3	6.25%	1	2	
Tumor stage at diagnosis						0.537
	T1	21	43.75%	15	6	
	T2	27	56.25%	17	10	
Size at diagnosis						0.685
	a <5 cm	25	52.08%	17	8	
	B >5 cm	21	43.75%	13	8	
	x: unavailable	2	4.17%	2	0	
Nodal stage at diagnosis						0.770
	N0	31	64.58%	20	11	
	N1	17	35.42%	12	5	
IRS group						0.849
	I	0	0%			
	IIa	3	6.25%	2	1	
	IIb	0	0%			
	IIc	0	0%			
	IIIa	38	79.17%	26	12	
	IIIb	7	14.58%	4	3	
	IV	0	0%			
Tumor location						0.279
	Orbit	3	6.25%	3	0	
	Non-parameningeal	15	31.25%	8	7	
	Parameningeal	30	62.5%	21	9	
Aggressiveness patterns for PM tumors (*n* = 30)
	Cranial nerve palsy	10	33.33%	7	3	1.000
	Skull base erosion	21	70%	15	6	1.000
	Intracranial extension	6	20%	4	2	1.000
Secondary resection of primary tumor						0.306
	Yes	26	54.17%	19	7	
	No	22	45.83%	13	9	
Nodal secondary surgery						0.036
	Yes	13	27.08%	12	1	
	No	35	72.92%	20	15	
Radiation therapy						1.000
	Yes	43	89.58%	29	14	
	No	5	10.42%	3	2	

*ARMS, alveolar rhabdomyosarcoma; TF, fusion transcript; PM, parameningeal. Bold text indicates a statistically significant difference*.

**Figure 4:**
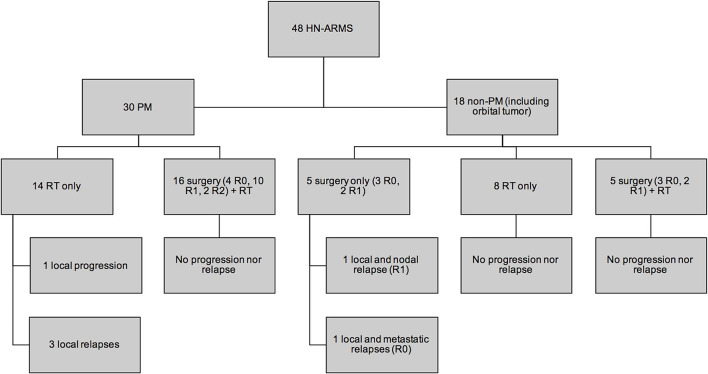
Local control according to the local treatments (surgery and radiation therapy).

**Figure 5:**
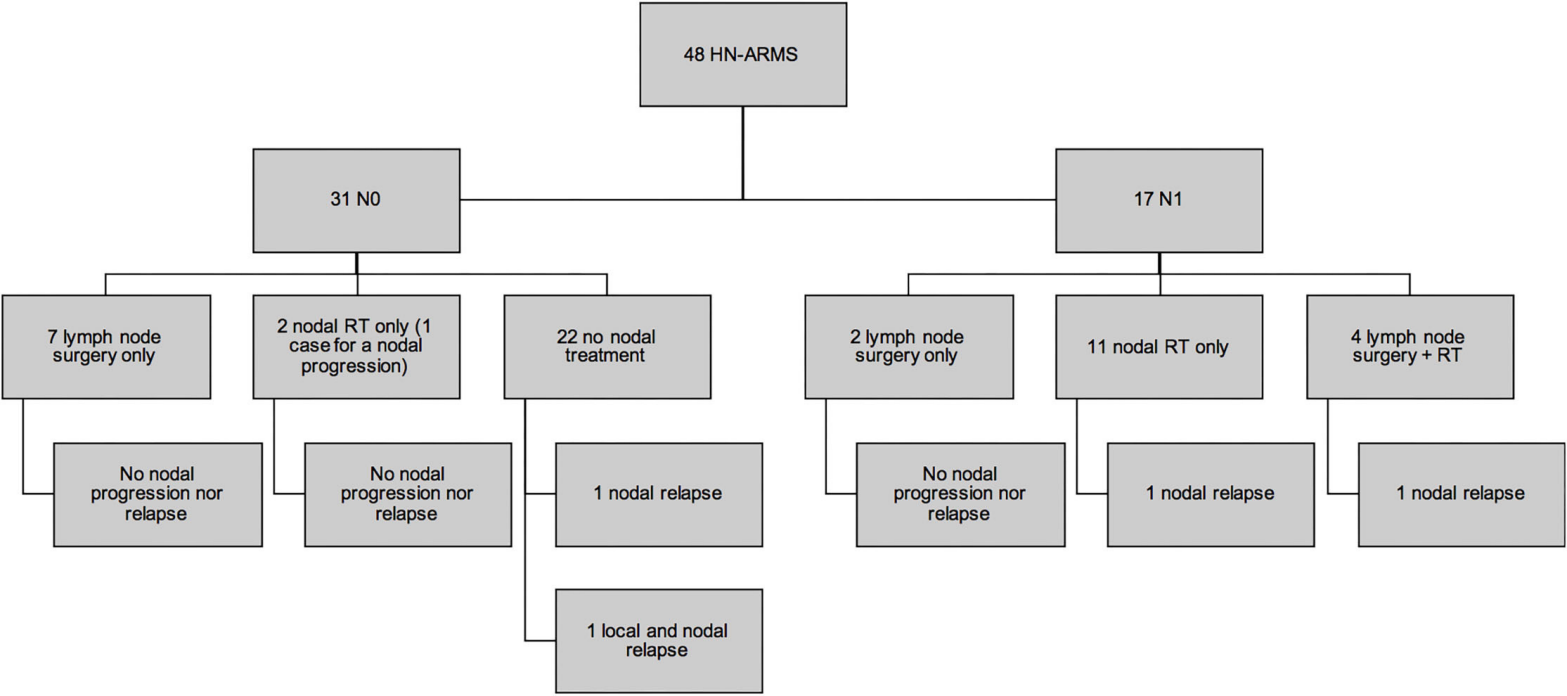
Nodal control according to the lymph node chain treatments (surgery and radiation therapy).

The existence of LN surgery was the only therapeutic modality associated with a lower risk of event occurrence in univariate analysis (Fisher's test exact, *p* = 0.036). No patient who had a secondary nodal surgery presented with metastatic relapse compared to 7 patients out of 35 (20%) without secondary LN surgery. No relapse was observed in patients who underwent both secondary surgery and postoperative RT (Figure 4). The two N1 patients who underwent LN dissection without RT had no nodal relapse (Figure 5).

### Survival Analysis

**Figure 6:**
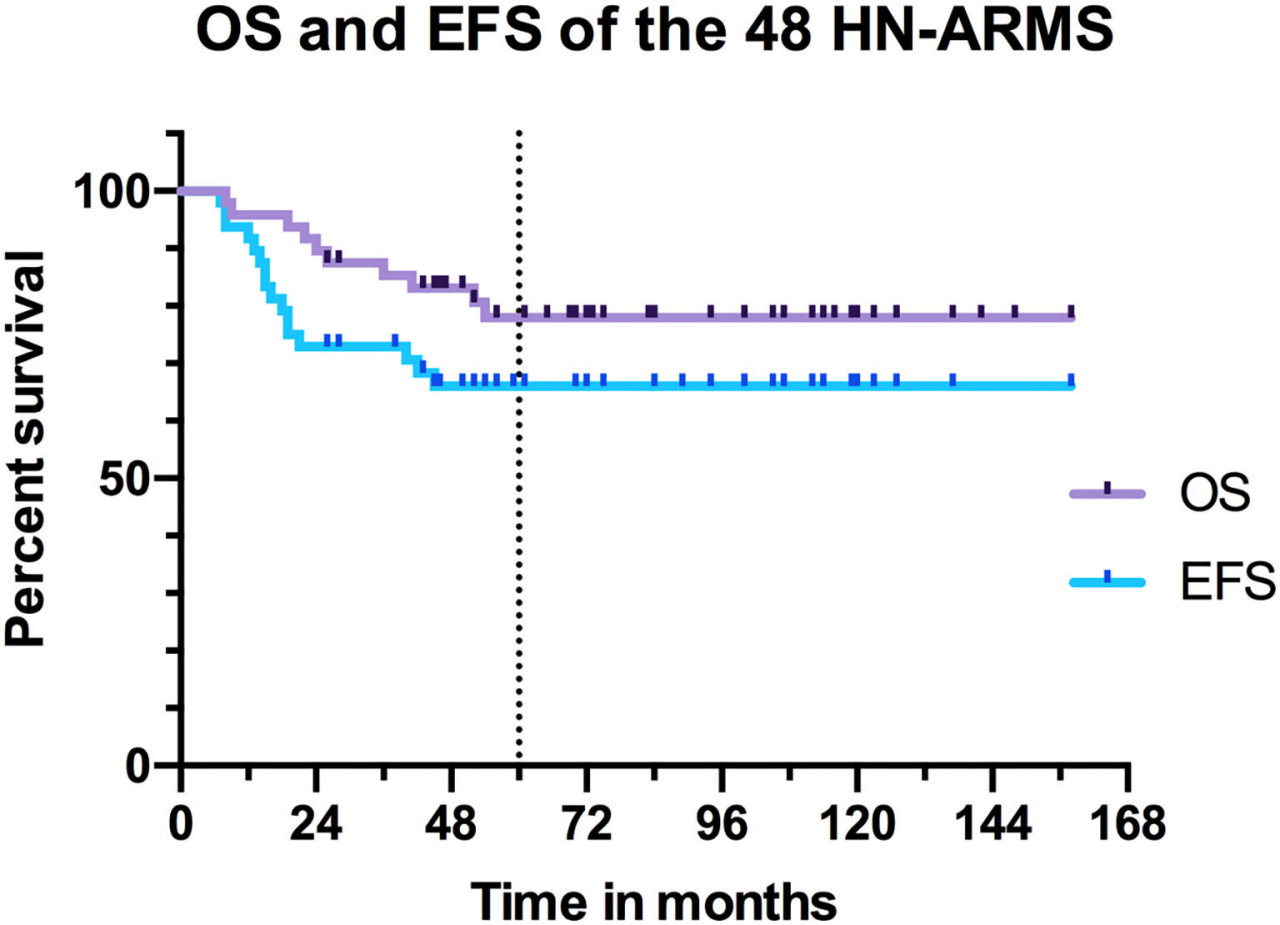
Overall survival and event-free survival of the 48 patients with head and neck alveolar rhabdomyosarcoma (HN-ARMS).

Thirty-eight patients (79.2%) were alive at the last visit date, and 10 patients (20.8%) were deceased. For alive patients, the median follow-up was 7.4 years (standard deviation 3.2 years; minimum 2.2 years; maximum 13.2 years). Of the 32 patients on first complete remission, 22 had at least 5 years of follow-up; median follow-up was 6.1 years (standard deviation 3.1 years; range 2.2–13.2 years). The 6 patients on second complete remission had at least 5 years of follow-up; median follow-up was 9.8 years (standard deviation 2.4 years; range 6.1–11.8 years). The 5-year OS was 78% [95% confidence interval (95% CI), 63–88%]. The 5-year EFS was 66% (95% CI, 51–78%) (Figure 6).

**Figure 7:**
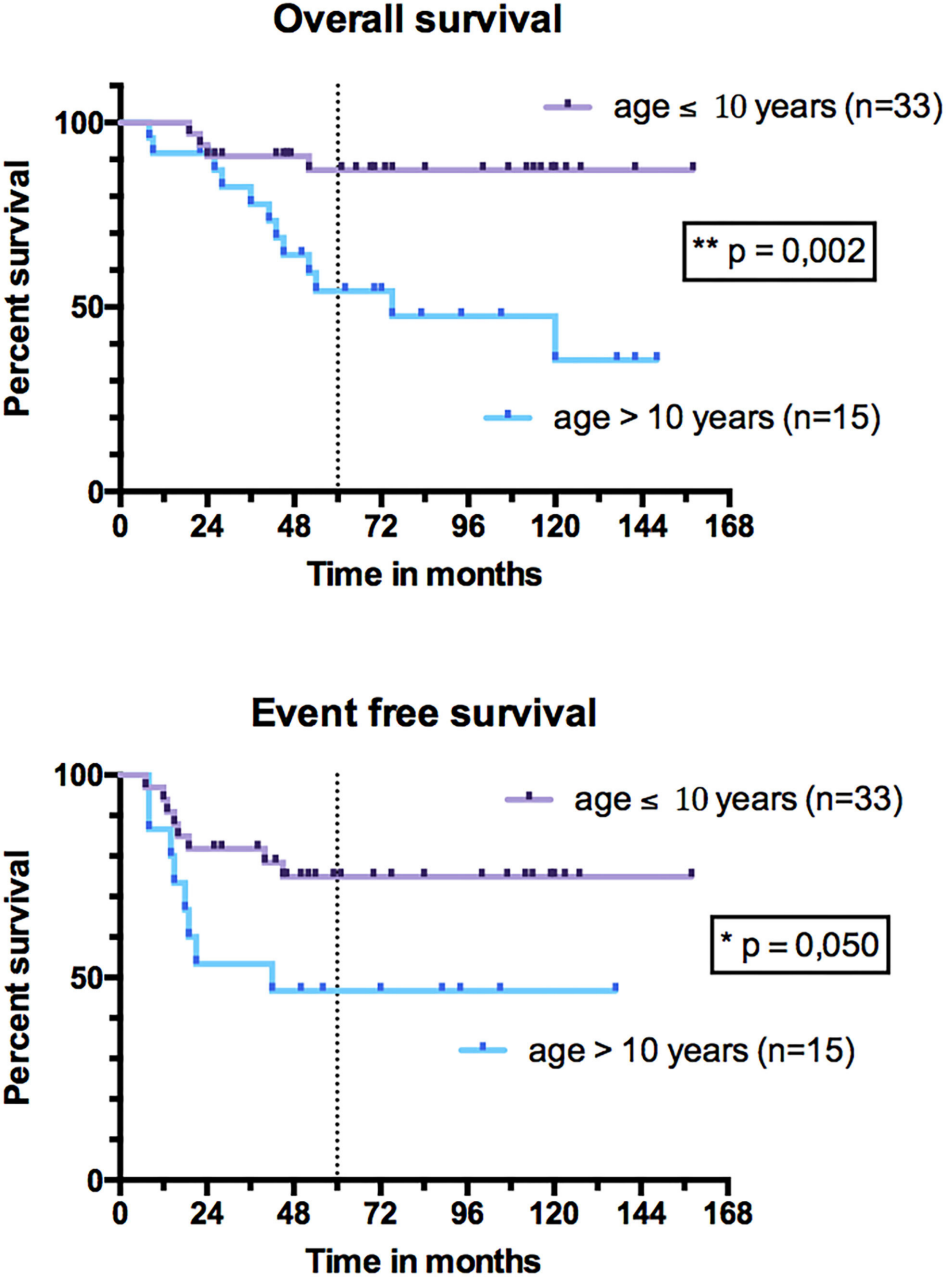
Overall survival and event-free survival of patients under 10 years and patients over 10 years.

**Figure 8:**
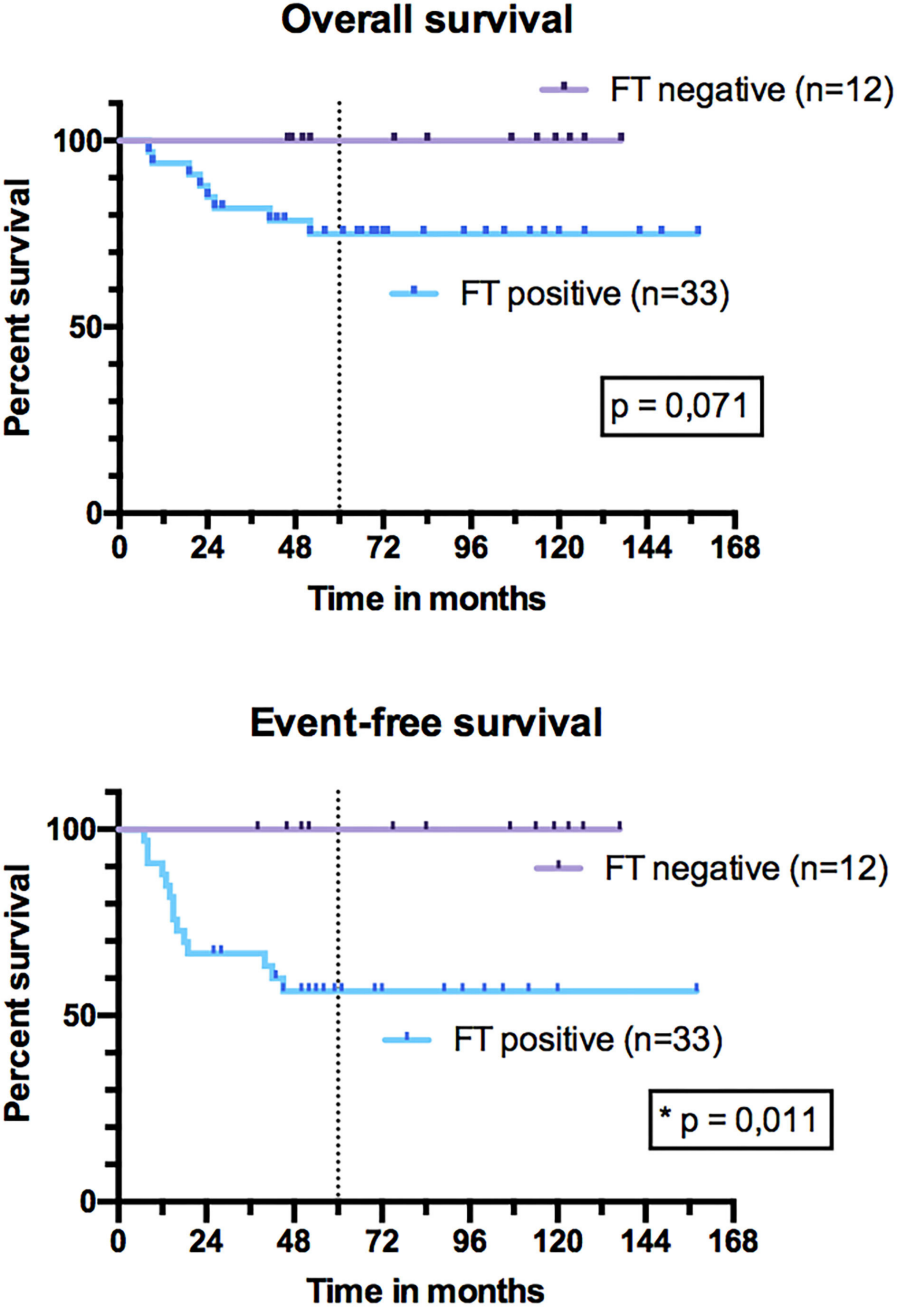
Overall survival and event-free survival of patients with fusion transcript (FT) expression and without FT expression.

In univariate analysis, sex, tumor and nodal stage, size, IRS stage, initial tumor location, resection margins, and radiation therapy did not influence OS and EFS (Table 5). Patients under 10 years had better OS than patients older than 10 years (*p* = 0.002) and better EFS (*p* = 0.050) (Figure 7). The 5-year OS was 87% (95% CI, 69–95%) for patients under 10 years of age and 54% (95% CI, 32–72%) for patients older than 10 years. The 5-year EFS was 75% (95% CI, 56–87%) in those under 10 years of age and 47% (95% CI, 21–69%) in those older than 10 years. Patients with tumors expressing a PAX3/PAX7-FOXO1 FT had an OS of 75% (95% CI, 56–87%) vs. 100% for those not expressing it (*p* = 0.071) (Figure 8). Patients with FT-negative tumors had significantly better EFS than those expressing a FT with 100% 5-year EFS vs. 56% (95% CI, 38–72%) (*p* = 0.011).

**Table 5:** Univariate analysis of 5-year OS and EFS according to patients and tumors' characteristics and treatment modalities for the 48 HN-ARMSs.

**Univariate analysis**		**5-year overall survival (CI)**	***p*** **-value**	**5-year event-free survival (CI)**	***p*** **-value**
Sex			0.988		0.993
	Male	78% (59–89%)		66% (48–80%)	
	Female	79% (48–93%)		64% (33–84%)	
Age (years)			0.002		**0.050**
	≤10	87% (69–95%)		75% (56–87%)	
	>10	54% (32–72%)		47% (21–69%)	
Tumor stage			0.352		0.580
	T1	84% (59–95%)		70% (44–85%)	
	T2	73% (51–86%)		63% (42–78%)	
Nodal stage			0.701		0.702
	N0	80% (60–90%)		64% (44–78%)	
	N1	75% (46–90%)		70% (42–86%)	
Size (cm)			0.283		0.667
	a ≤5	81% (57–93%)		66% (43–82%)	
	b >5	71% (47–86%)		62% (38–79%)	
IRS stage			0.291		0.801
	IIa	100%		67% (5–95%)	
	IIIa	81% (65–91%)		68% (50–80%)	
	IIIb	51% (12–81%)		57% (17–84%)	
Tumor location			0.622		0.532
	Non-PM (including orbital)	82% (54–94%)		58% (31–77%)	
	PM	76% (56–88%)		70% (50–83%)	
Local aggressiveness for PM			0.809		0.961
	Skull base erosion	76% (52–89%)		71% (47–86%)	
	No skull base erosion	76% (33–94%)		67% (28–88%)	
			0.413		0.661
	Intracranial extension	67% (19–90%)		67% (19–90%)	
	No intracranial extension	78% (54–90%)		71% (48–85%)	
			0.452		0.797
	Cranial nerve palsy	70% (33–89%)		70% (33–89%)	
	No cranial nerve palsy	78% (52–91%)		70% (45–85%)	
FT expression			0.071		**0.011**
	Yes	75% (56–87%)		56% (38–72%)	
	No	100%		100%	
Secondary resection of primary tumor			0.348		0.323
	Yes	84% (62–94%)		72% (51–86%)	
	No	71% (47–86%)		58% (35–76%)	
			0.176		**0.036**
	PM ARMS operated	88% (59–97%)		88% (59–97%)	
	PM ARMS non-operated	63% (32–83%)		50% (23–72%)	
			0.711		0.281
	Non-PM ARMS operated	77% (34–94%)		44% (12–73%)	
	Non-PM ARMS unoperated	88% (39–98%)		73% (28–93%)	
Resection margins			0.277		0.696
	R0	67% (27–88%)		70% (33–89%)	
	R1	93% (59–99%)		71% (41–88%)	
	R2	100%		100%	
Nodal secondary surgery			0.210		**0.034**
	Yes	92% (57–99%)		92% (57–99%)	
	No	73% (54–85%)		56% (38–71%)	
RT			0.958		0.768
	Yes	78% (62–88%)		67% (51–79%)	
	No	75% (13–96%)		53% (7–86%)	

*CI, confidence interval; PM, parameningeal; ARMS, alveolar rhabdomyosarcoma; FT, fusion transcript; RT, radiation therapy. Bold text indicates a statistically significant difference*.

**Figure 9:**
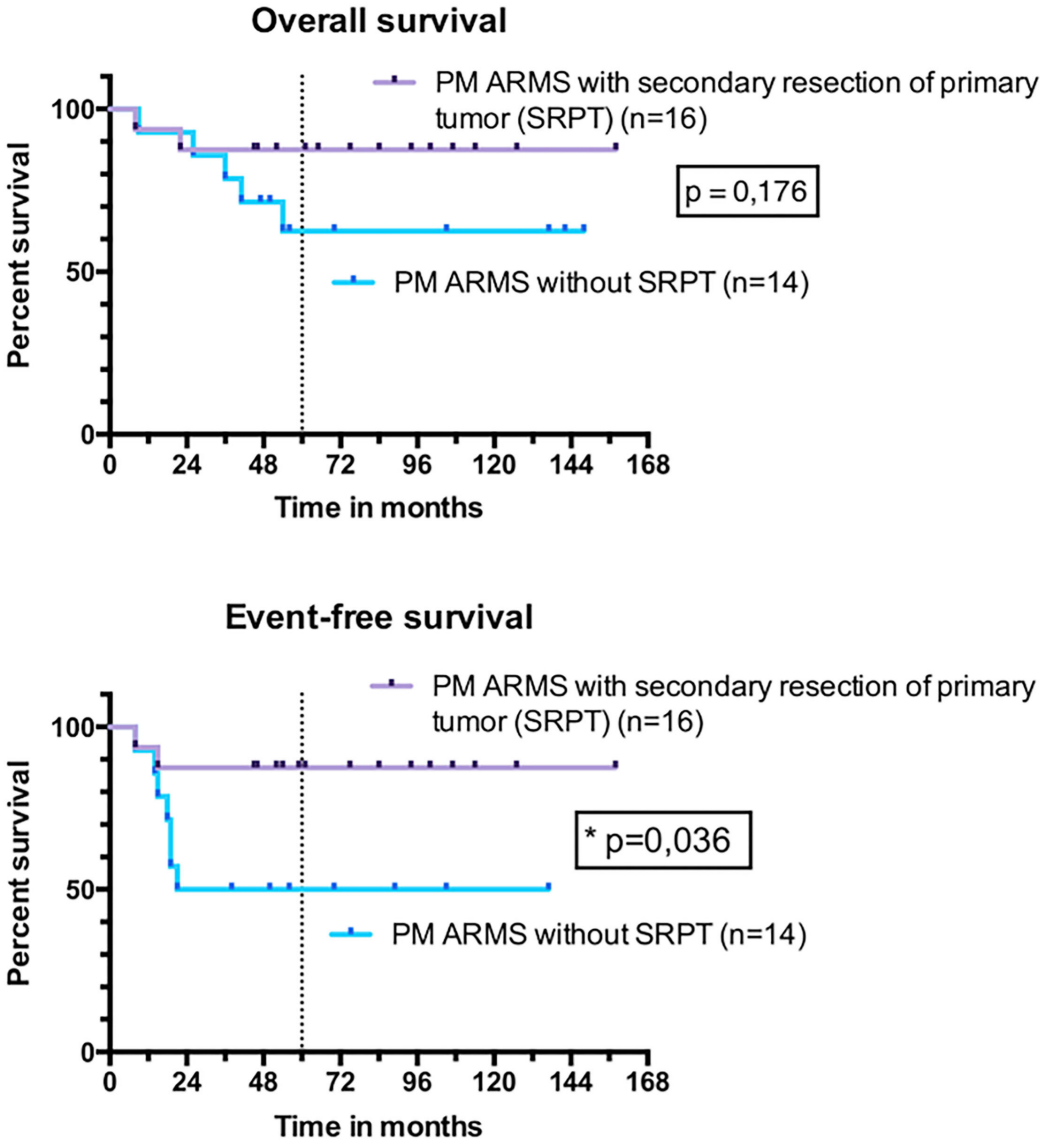
Overall survival and event-free survival of patients with parameningeal alveolar rhabdomyosarcoma (PM ARMS) who had undergone a secondary resection of primary tumor and patients who did not.

**Figure 10:**
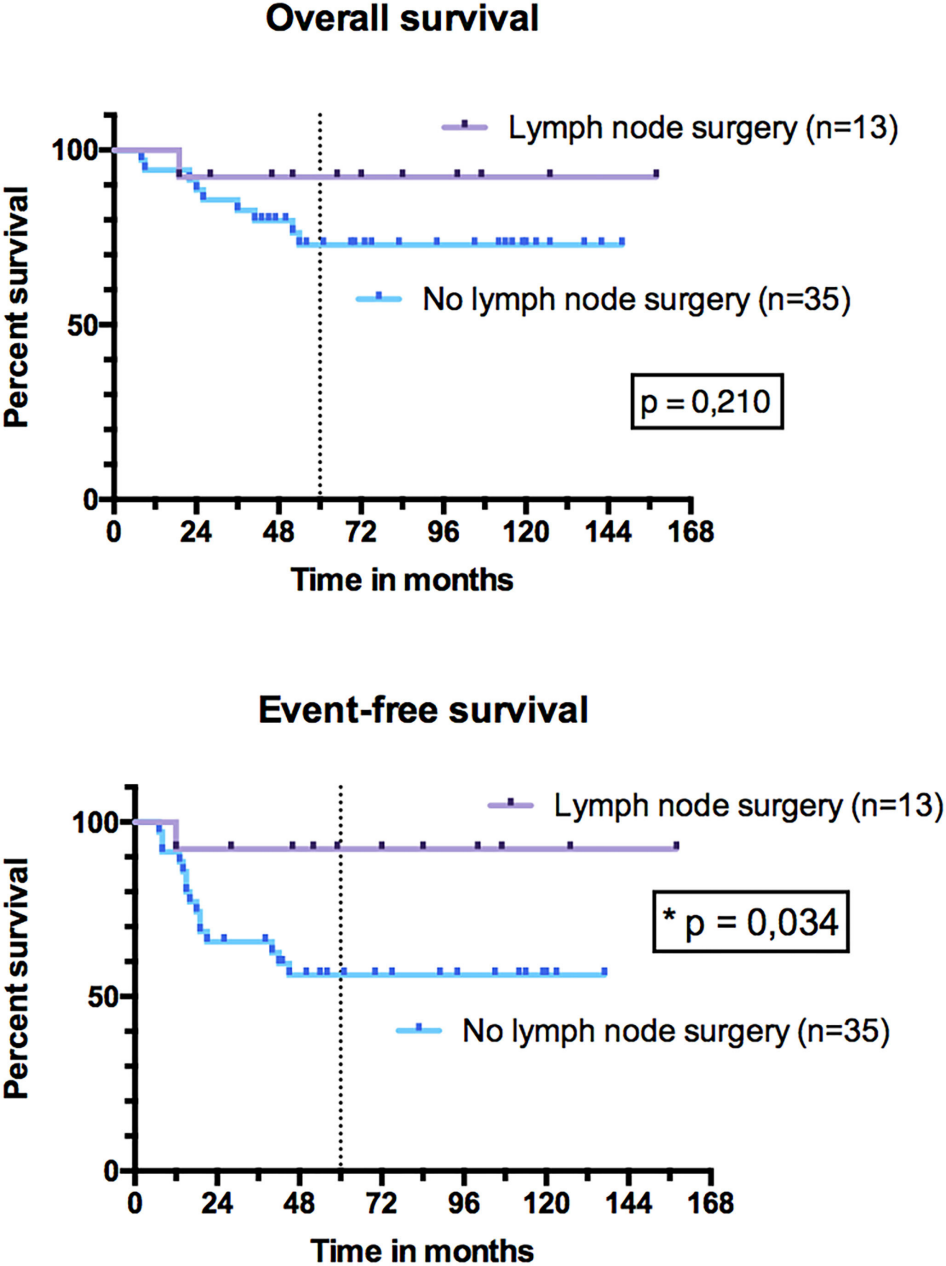
Overall survival and event-free survival of patients with head and neck alveolar rhabdomyosarcoma (HN-ARMS) who underwent a lymph node dissection compared to those who did not.

For PM ARMS, the 5-year OS was 88% (95% CI, 59–97%) for patients who underwent SRPT vs. 63% (95% CI, 32–83%) in those who did not (Figure 9) (*p* = 0.176). The EFS was significantly higher (*p* = 0.036) in patients with PM ARMS who underwent SRPT compared to those who did not. The 5-year EFS in operated PM tumors was 88% (95% CI, 59–97%) vs. 50% (95% CI, 23–72%) in non-operated patients. The 5-year OS was 92% (95% CI, 57–99%) for patients who underwent LN surgery vs. 73% (95% CI, 54–85%) in those who did not (*p* = 0.210) (Figure 10). EFS was significantly higher for patients who underwent LN surgery with 5-year EFS at 92% (95% CI, 57–99%) compared to those who did not with 5-year EFS at 56% (95% CI, 38–71%) (*p* = 0.034).

## Discussion

RMSs, although rare in the general population, are among the most common tumors in children. The alveolar histological subtype is associated with a poorer prognosis that justifies a high burden of therapy. Surgery is theoretically reserved for patients with resectable negative margin tumor and remains a carcinological, functional, and aesthetic challenge.

### Characteristics of the Population and of the Tumors

Characteristics of our population are comparable to those of Ludmir et al. (6) and Radzikowska et al. (21) series (with 14 pediatric HN-ARMS and 36 pediatric HN-RMS cases, respectively) concerning the sex ratio (respective percentages of boys in these 3 series: 69, 64, 67%) and for the median age (respective values for these 3 series: 6, 7, and 7 years). Initial tumor location was PM in 62.5% of the cases in our series, which is comparable to the 57% observed by Ludmir et al. (6) and to the 67% observed by Radzikowska et al. (21). Intracranial extension of PM ARMS was present in 20% of our cases and in 14.3% of cases in the series by Ludmir et al. (6). The percentage of tumors expressing a PAX3-PAX7/FOXO1 FT was 68.75% in our cohort, which is similar to the 67% observed by Bradley et al. (22) in a series of 24 pediatric PM ARMS. The proportion of LN involvement at diagnosis (N1) was slightly lower in our study (35.4%) compared to that (42.9%) of Ludmir et al. This difference could be explained by the exclusion from our study of patients with metastatic disease at the time of diagnosis. Initial assessment is essential, in particular, to determine the nodal extension of the disease. Several studies suggest the benefit of performing a PET-CT at the time of diagnosis given its high sensitivity and specificity (23–26). However, at variance with these studies, and in accordance with the publication of Ludmir et al. (6), we did not observe any association between the inclusion of a PET-CT in the diagnostic workup and patients' initial N status.

### Survival and Prognostic Factors

The 5-year OS and EFS of our series (respectively, 78 and 66%) appear to be higher than those observed by Dantonello et al. (27) in a series of 235 pediatric ARMS of any location (58 and 47%, respectively). This difference could suggest a better prognosis for HN-ARMS compared to other locations. Our 5-year OS and EFS are higher than the series of 14 pediatric HN-ARMS (respectively, 45 and 25%) of Ludmir et al. (6). This could be explained by the higher percentage of FT-negative tumor in our series (25%) compared to that (14%) of Ludmir et al. Indeed, in univariate analysis, tumor expression of a PAX3/PAX7-FOXO1 FT was a significant risk factor for relapse (*p* = 0.004). This is consistent with other large series, which suggest using the FT as a prognostic factor to reassign FT-negative patients to a lower treatment group. Children under 10 years of age had a better prognosis in our series, with 5-year OS and EFS of 87 and 75%, respectively. In univariate analysis, age over 10 years was an event risk factor (*p* = 0.048). This observation was also made by several authors for RMS of any location (27, 28) and more specifically for HN-ARMS (15). Considering these risk factors, therapeutic burden may be adapted—with less systematic radiotherapy in case of adequate and complete SPRT performed by a referent surgical team—for patients under 10 years of age. In a series of 140 localized non-PM RMS including 40 RMSA from 1984 to 2004, Orbach et al. (29) observed a 5-year OS of 66% and a 5-year EFS of 51%. This difference with the OS and EFS observed in our series seems all the more significant as the one by Orbach et al. (29) includes a majority of ERMSs that have a better prognosis than ARMS. Neither can this difference be explained by patients' ages [median age 5 years in the series by Orbach et al. (29) and 6.2 years in ours] or by the percentages of operated patients (64 and 54%, respectively). These differences in OS and EFS could be linked to the improvement in the quality and the tolerance of the systemic treatment, as well as to a greater efficiency of the local and regional treatment with conformational and proton beam radiation therapy and the development of free flaps, allowing large surgical resections for “ghost surgeries.” Several authors suggest that OS and EFS are better in patients who underwent surgical resection in addition to treatment with chemotherapy and RT (15, 30–32). Multidisciplinary approach including “ghost surgery” for PM sarcoma is feasible and yields promising local control. This strategy may help to avoid RT or limit the RT field for young children and improve local control for these unfavorable PM sites (33). Indeed, with RT only, PM RMSs have bad outcomes, with survival of only about 60% in the more recent studies, which has to be warranted by future studies. For the 30 PM-ARMS of our series, the EFS was significantly better after SRPT (*p* = 0.036). This difference in favor of surgery was not observed for non-PM tumors, nor for all locations combined, nor for OS. Patients under 10 years of age, with better prognosis, were significantly more operated on (*p* = 0.002) in the hope of avoiding radiation therapy in some younger children. This might partly explain the difference in favor of surgery. In our series, most primary surgeries were performed without respecting the usual oncologic surgery principles (safety resection margins), explaining that none of the patients were IRS I. However, there was no significant difference in OS and EFS as a function of the IRS group (*p* = 0.291 and *p* = 0.801, respectively). Despite this absence of correlation between incomplete PRPT and events, PRPT is not indicated in the management of HN-ARMS if a negative margin resection is not possible because it compromises the evolution assessment.

In our series, 4 resections needed a free flap reconstruction, most often requiring a multidisciplinary team specialized in this kind of complex surgical procedure, and 13 SRPTs were mutilating (3 facial paralysis, 5 infratemporal fossa resections, 7 maxillary resections, and 2 mandibular resections). The large proportion of patients who underwent SRPT (54%) as a core of initial therapy can be explained by improvements in perioperative management as well as surgical techniques in children in the last few years. Postoperative morbidity has also been reduced—thanks to the development of combined minimally invasive endoscopic and transcranial or transfacial approaches and free-flap reconstructive possibilities, which limit functional and cosmetic sequelae (34–36). In the retrospective study of 92 HN-RMS (both ERMS and ARMS) by Dombrowski et al. (16), surgery was associated with a reduced risk of mortality after adjusting for TNM staging and location of the tumor (*p* = 0.05). Furthermore, in our series, the quality of the resection assessed by the resection margins (R0, R1, or R2) was not correlated with OS and EFS. Yunteng et al. (37) also made this observation on his series of 51 HN-RMS (*p* = 0.86). This might be due to the fact that RT, which is proposed in most cases after surgery, helps control any postoperative tumor remnant, especially in anatomical areas where it is impossible to operate with safe resection margins such as cavernous sinus or skull base foramina. In our series, only 5 patients did not receive RT because of long-term sequelae of radiation for very young children.

In our series, performing LN dissection was associated with better EFS (*p* = 0.034). However, patients who underwent an LN secondary surgery had a lower rate of LN relapse (7.7%) than those who did not (11.4%), but the difference concerned more metastatic relapses (0% in case of LN secondary surgery and 20% in the absence of it). We suggest that LN dissection does not increase so much the burden of therapy, especially if it is carried out at the same time as secondary surgery of the primary tumor. More importantly, in the future, negative neck dissection (pN0) could justify to not perform radiation therapy on LN areas. In their series of 14 HN-ARMSs, Ludmir et al. (6) observed 57% of LN relapses vs. 8.3% in our series. These two series are mainly different in terms of rates of N1 patients at diagnosis, rates of FT-positive tumors as described before, and therapeutic strategy [SRPT for 26 patients in our series compared to medical therapy with chemotherapy and RT in the series by Ludmir et al. (6)], which could explain the difference in LN relapse rates. No difference in OS and EFS was brought out according to RT. This could be explained by the small size of our cohort and the low number of patients who did not receive RT. Indeed, in a meta-analysis including 1,105 PM RMSs, Merks et al. (38) found a poorer 10-year OS for non-irradiated patients (40.8%) than for irradiated patients (68.5%).

### Limitations of the Study

Although our population is histologically homogeneous, the proportion of tumors not expressing PAX3/PAX7-FOXO1 fusion (25%) may be a bias for the analysis of ARMS survival. Indeed, it is now well-recognized that about 20–25% of ARMSs do not express FOXO1 fusion conferring specific clinic and biologic characteristics with inferior outcomes (39). Despite the difference of prognosis of these 2 molecular subtypes, FT-positive and FT-negative tumors, our cohort reflects the real-life experience in France. Moreover, the RMS2005 study recommended RMS prognostic stratification and therapeutic decision based on histology only. Of course, in the current era, FOXO1 fusion instead of histology is used even if a minority of tumors are still histologically classified as “true” ARMS lacking the canonical PAX-FOXO1 fusion but had new molecular alterations (40). In addition, the small size of the population (*n* = 48 patients) limits the statistical test power. Diversity of locations in HN region and of therapeutic strategies complicates the results' interpretation. Finally, the morbidity of the various treatments has not been exhaustively studied and could have been underevaluated. In conclusion, management of HN-ARMS is an oncological, radiotherapeutic, and surgical challenge. The initial staging is essential to best adapt the systemic and locoregional treatment. SRPT could improve the EFS of PM tumors, and LN surgery could improve EFS. SRPT must respect the principles of oncologic surgery while limiting mutilating procedures in order to preserve the quality of life of patients. Further larger international analyses of HN ARMS, including not only pediatric oncologists but also HN surgeons and RT physicians, are needed to confirm these findings.

## Data Availability Statement

The original contributions presented in the study are included in the article/supplementary material, further inquiries can be directed to the corresponding authors.

## Ethics Statement

All participating Centres were required to obtain written approval from their local authorities and Ethical Committees, as well as written informed consent from patients or their parents or legal guardians.

## Author Contributions

All authors listed have made a substantial, direct, and intellectual contribution to the work and approved it for publication.

## Conflict of Interest

The authors declare that the research was conducted in the absence of any commercial or financial relationships that could be construed as a potential conflict of interest.

## Publisher's Note

All claims expressed in this article are solely those of the authors and do not necessarily represent those of their affiliated organizations, or those of the publisher, the editors and the reviewers. Any product that may be evaluated in this article, or claim that may be made by its manufacturer, is not guaranteed or endorsed by the publisher.
